# Performance evaluation of different albumin assays for the detection of analbuminemia

**DOI:** 10.1371/journal.pone.0300130

**Published:** 2024-03-06

**Authors:** Yi Zhang, Afsoun Abdollahi, Chaylen Andolino, Keigo Tomoo, Bailey M. Foster, Uma K. Aryal, Gregory C. Henderson

**Affiliations:** 1 Department of Nutrition Science, Purdue University, West Lafayette, IN, United States of America; 2 Purdue Proteomics Facility, Purdue University, West Lafayette, IN, United States of America; 3 Department of Comparative Pathobiology, Purdue University, West Lafayette, IN, United States of America; Kwame Nkrumah University of Science and Technology, GHANA

## Abstract

Analbuminemia is characterized by the near absence of albumin in the plasma. Different methods are available for measuring albumin levels, but they do not necessarily agree with one another. It is a concern that analbuminemic samples could be falsely characterized due to the incorrect estimation of albumin. The objective of the work was to evaluate the performance of different assays in detecting analbuminemia. Albumin knockout (Alb^-/-^) mouse plasma was used to test the suitability of different albumin assays for their ability to properly characterize extreme albumin deficiency. Bromocresol green (BCG), bromocresol purple (BCP), enzyme-linked immunosorbent assay (ELISA), liquid chromatography-tandem mass spectrometry (LC-MS/MS), and gel electrophoresis were tested. The LC-MS/MS assay exhibited broad coverage of the amino acid sequence of albumin and indicated 8,400-fold lower (*P*<0.0001) albumin expression in Alb^-/-^ than wildtype (WT), demonstrating its suitability for identifying extreme albumin deficiency. ELISA estimated albumin at 1.5±0.1 g/dL in WT and was below the detection limit in all Alb^-/-^ samples. Gel electrophoresis yielded consistent results with LC-MS/MS and ELISA. The BCG assay overestimated albumin with apparently appreciable albumin concentrations in Alb^-/-^ mice, yet the assay still indicated a significant difference between genotypes (Alb^-/-^, 1.2±0.05 g/dL, WT, 3.7±0.1 g/dL, *P*<0.0001). BCP drastically overestimated albumin and could not successfully identify the known analbuminemic phenotype of Alb^-/-^ mice. By using Alb^-/-^ plasma as a reference material and LC-MS/MS as a reference method, ELISA and gel electrophoresis appear appropriate for identifying analbuminemia, while BCG and BCP are not suitable. It is concluded that dye-binding assays should be avoided when extreme hypoalbuminemia or analbuminemia is suspected.

## Introduction

Albumin is the most abundant protein in the plasma of nearly all vertebrates [1]. The concentration of plasma albumin is used as a disease severity marker in various clinical conditions related to hepatology, nephrology and nutritional status [2]. Current albumin testing methods include dye-binding methods, immunoassays, high-performance liquid chromatography, electrophoresis and near-infrared spectroscopy [3]. Dye-binding methods, especially Bromocresol green (BCG) and Bromocresol purple (BCP), are the most employed options [3]. Despite the wide use of BCG and BCP, a body of literature describes potential inaccuracies in the results obtained by both [4–7]. Numerous reports have concluded that the BCG assay overestimated albumin concentration and that BCP was potentially more accurate. These conclusions had been based on a comparison to immunoassays (immunoturbidimetry or immunonephelometry) which have been viewed by some as the gold standard methods [8]. Importantly, the lack of agreement between BCG and immunoassay is even more profound in pathological plasma/serum [4, 9]. BCP provided closer results to the immunoassay reference method in patients with hypoalbuminemia and/or renal disease but has been reported to either underestimate or overestimate albumin in varying clinical sample types [6, 10–12]. There remains a lack of consensus regarding the utility of BCG and BCP assays, potentially due to the absence of proper control samples for quality assurance. In methodology assessment papers, assay types have been compared without the benefit of analyzing a well-characterized hypoalbuminemia reference material. If an assay were to detect appreciable albumin in a reference material that is known to be nearly albumin-free, this would indicate an evident concern about assay performance. Until now, such method suitability testing has not been proposed. Identifying this new and impactful practice could enhance our ability to determine albumin assay suitability for plasma from patients who are potentially analbuminemic. Such findings would provide evidence for choosing the proper method to measure albumin to facilitate proper clinical diagnosis and monitoring during treatment.

Congenital analbuminemia (CAA) is a rare autosomal recessive disorder in which patients express no albumin or only extremely low levels [13]. The diagnosis of CAA is typically by albumin protein measurements, often followed by confirmation through genetic testing [14]. A significant volume of case reports about CAA noted markedly higher albumin concentration values from BCG and BCP assays as compared to values determined by electrophoresis and immunoassays [14–17]. It seemed possible that exclusive use of a dye-binding assay to detect CAA would be inadequate. To verify that indeed BCG or BCP were overestimating albumin, a reference material known to be albumin-free (or nearly albumin-free) would have been needed. As the genetic basis for CAA is diverse, and a small amount of albumin could still be produced in some cases [13], it is not feasible to review data from CAA case reports to conclusively determine which assays were most appropriate for properly characterizing analbuminemia.

Plasma from the albumin knockout (Alb^-/-^) mouse model represents a potentially useful reference material to test the suitability of methods for detecting analbuminemia. This model was reported to have no detectable albumin expression by enzyme-linked immunosorbent assay (ELISA) and gel electrophoresis [18, 19] and an approximately 20,000-fold reduction in albumin expression as determined by mass spectrometry (MS) [20]. With the high sensitivity of the mass spectrometry method in detecting a very small amount of albumin in the gene knockout mice, it could be reasonable to approximate this low but MS-detectable level of albumin to zero. Furthermore, because the mouse model is commercially available (Jackson Laboratory, Bar Harbor, ME, USA), it represents an abundantly accessible resource in comparison to the small amount of human CAA plasma available to investigators. In Alb^-/-^ mice, albumin expression silencing is achieved by a single base deletion that results in a frameshift followed by a premature stop codon 55 amino acids downstream of the encoded frameshift, likely leading to rapid degradation of the highly truncated protein product [18]. Thus, Alb^-/-^ mouse plasma could potentially act as an albumin-free reference material to be used for assessing different albumin assay approaches.

There were two goals of this study. The first was to test the feasibility of using Alb^-/-^ mouse plasma as a negative control by confirming the absence of intact albumin protein and major albumin fragments. The second goal was to use this type of plasma as a negative control to evaluate the suitability of albumin assays for the detection of analbuminemia. To the best of our knowledge, this is the first time that a proper negative control has been used to benchmark the performance of albumin assays. From this perspective, we hope to raise awareness of the inappropriate use of certain albumin testing methods in clinical practices and to reduce the risk of erroneous characterization of patients. We hypothesized that BCG would overestimate plasma albumin levels while the other assay types would be more accurate for identifying analbuminemia.

## Materials and methods

### Animals

All experiments were approved by the Purdue University Animal Care and Use Committee (protocol number: 1911001983). We studied male Alb^-/-^ mice (Jackson Laboratory strain # 025200) and wildtype (WT, Jackson Laboratory strain # 000664) mice, both on the C57BL/6J background; readers are referred to the original publication on this Alb^-/-^ model for the details of its initial development [18]. Mice were housed in individually ventilated cages with up to 5 mice per cage. All the mice were allowed ad libitum access to water and a chow diet. Mice were free of apparent illness at euthanasia, which was conducted at ~8 weeks old by carbon dioxide inhalation, followed by exsanguination via cardiac puncture. Blood was collected into ethylenediaminetetraacetic acid tubes and plasma was separated via centrifuge and then stored at -80°C until analysis. For each albumin measurement assay, a sample size of 5–8 mice was used. The plasma samples were analyzed individually, rather than being pooled, in order to allow biological variability to be represented in the datasets.

### LC-MS/MS

We previously reported data on plasma proteins in Alb^-/-^ mice using a mass spectrometry-based proteomics approach [21]. In that report, the quantitative results for albumin were not presented. Here, for methods evaluation, the label-free quantitation (LFQ) results for plasma albumin are presented from that data set. Proteins from an equal volume of each plasma sample (1 μL) were reduced using 10 mM dithiothreitol and alkylated with 20 mM iodoacetamide before digestion with 2 μg of Trypsin/Lys-C Mix (Promega, Madison, WI, USA). Samples were cleaned using MicroSpin^TM^ C18 reversed phase spin columns (The Nest Group, Southboro, MA, USA). Cleaned peptides were dried using a vacuum centrifuge, resuspended in 3% acetonitrile/0.1% formic acid, and loaded into a Dionex UltiMate 3000 liquid chromatography system coupled with an Orbitrap Fusion Lumos Mass Spectrometer (Thermo Fisher Scientific, Waltham, MA, USA). Reversed-phase peptide separation was accomplished using a trap column (300 mm ID × 5 mm) packed with 5 mm 100 Å PepMap C18 medium coupled to a 50-cm long × 75 μm inner diameter analytical column packed with 2 μm 100 Å PepMap C18 silica (Thermo Fisher Scientific). The data were processed using MaxQuant software against the UniProtKB Mus musculus protein database and quantified by the LFQ method. An LC-MS/MS assay was used to verify the complete absence or nearly complete absence of albumin in the Alb^-/-^ mice. All peptides associated with albumin and peptides unique to murine albumin were selected for possible detection in all mouse samples.

### Gel electrophoresis

Plasma samples were diluted 40-fold with Laemmli Sample Buffer (Biorad, Hercules, CA, USA) and β-mercaptoethanol. 20 μL of diluted plasma were then loaded and separated by sodium dodecyl sulfate-polyacrylamide gel electrophoresis (SDS-PAGE) on a 4–15% Tris-HCl precast gel (BioRad), with human serum albumin as a reference. The gel electrophoresis was conducted with a voltage set at 100 V for 10 min and at 200 V for approximately 45 min until the loading dye reached near the bottom of the gel. The gel was then stained by Coomassie Brilliant Blue R-250 Staining Solution (Biorad) for one hour, followed by destaining with Coomassie Brilliant Blue R-250 Destaining Solution (Biorad). The resulting gel was then scanned using an Odyssey CLx Imager (LI-COR Biosciences, Lincoln NE, USA).

### BCG and BCP

The BCG assay kit was purchased from Millipore-Sigma (Burlington, MA, USA, MAK124). As per manufacturer instructions, plasma samples and the bovine albumin standard were diluted two-fold in ultrapure water. 5 μL of the diluted plasma sample or standard was added into each well in duplicate, followed by the addition of 200 μL of BCG reagent. After incubation for 5 minutes at room temperature, absorbance at 620 nm was obtained and values were quantified based on the standard curve. The BCP assay kit was initially purchased from Millipore-Sigma (MAK125). When it became clear that the BCP kit performance was unfavorable, another BCP kit was purchased from Abcam (Boston, MA, USA, ab272526) to confirm the prior finding. For BCP kits, as per manufacturer instructions, samples were diluted two-fold, and 20 μL of the diluted samples were used for measurement, with calibration using standard curves and absorbance measurement at 610 nm.

### ELISA

A mouse albumin ELISA kit from Abcam (ab207620) was used in this study. The standard curve was generated using the provided mouse albumin protein. Plasma samples were diluted 10 million-fold in wash buffer and dilution buffer according to the manufacturer’s instructions. 50 μL of standard or diluted samples were used for the assay with the addition of 50 μL of antibody cocktail. One-hour incubation was performed at room temperature. Subsequently, wells were washed three times, and the reactions were completed using development solutions and stop solutions. Both standards and samples were loaded in duplicate. Results were obtained at 450 nm.

### Statistical analysis

Data are presented as means ± standard error. The results were analyzed by t-test with JMP version 16 (SAS Institute Inc., Cary, NC, USA) with a two-sided *P* ≤ 0.05 considered statistically significant.

## Results and discussion

### LC-MS/MS

**Fig 1:**
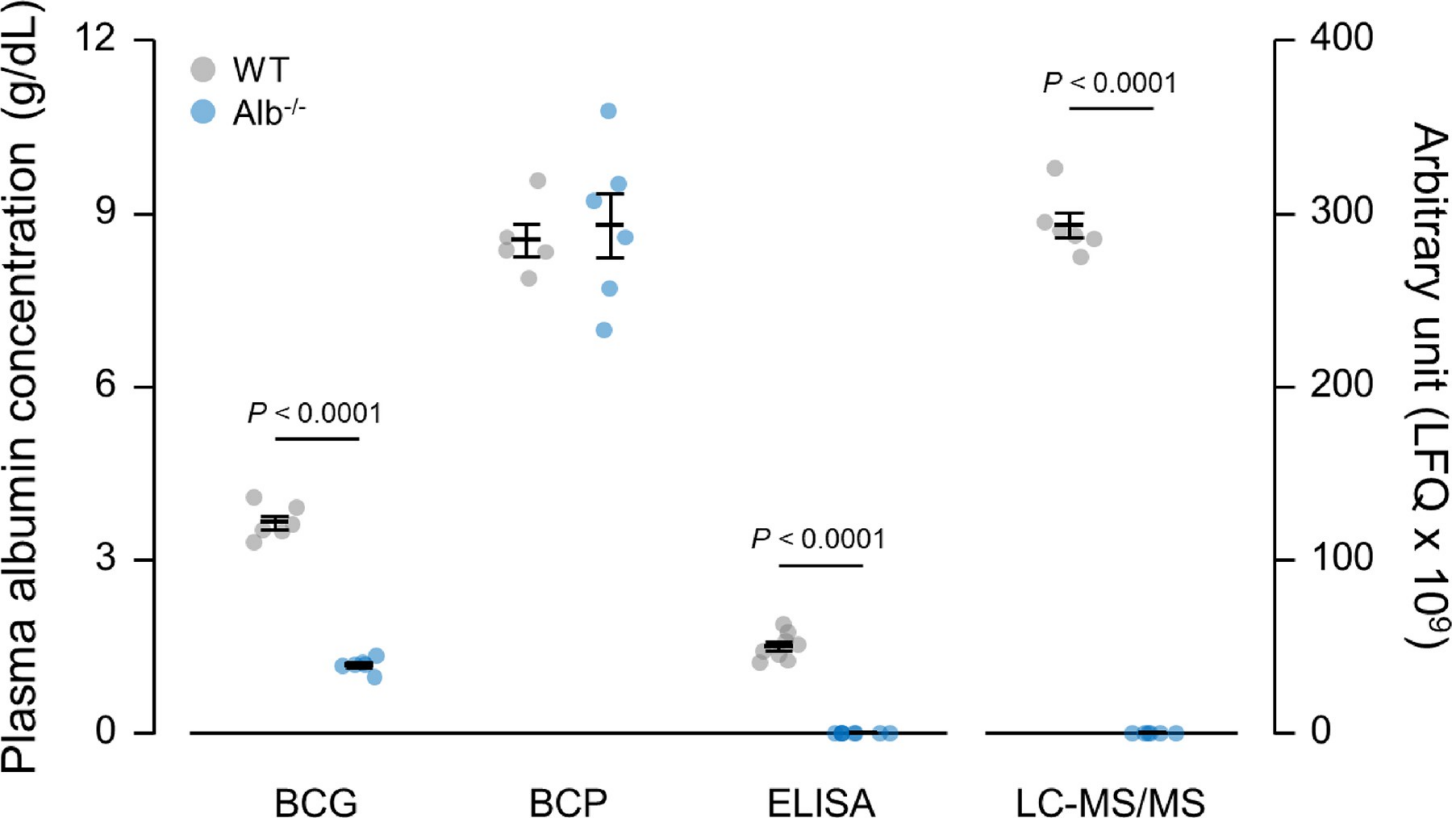
Plasma albumin level measurements by BCG, BCP, ELISA and LC-MS/MS. Analysis by student t-test. *n* = 5–8 per group. Data are presented as the mean ± standard error.

**Fig 2:**
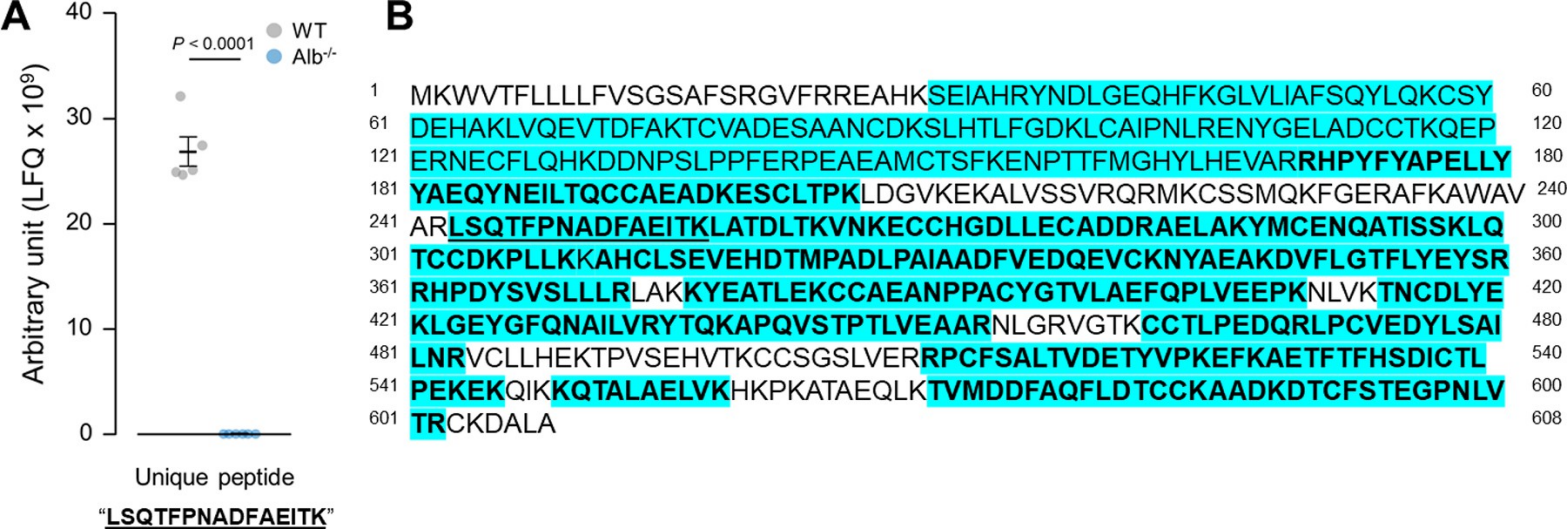
Albumin sequence obtained by LC-MS/MS. A). Unique peptide “LSQTFPNADFAEITK” levels in Alb^-/-^ and WT mice. Analysis by student t-test. *n* = 6 per group. Data are presented as the mean ± standard error. B). Amino acid sequence coverage for albumin in proteomics analysis. Blue highlights: all peptides identified in WT mice, 482 amino acids, 79% coverage. Bold letters: unique peptides identified in all WT replicates, 342 amino acids; 56% coverage. Underlined letters: unique peptides identified in all albumin Alb^-/-^ replicates, 15 amino acids; 2.5% coverage. Unique peptides: peptides that are unique to murine albumin, with no overlap with other protein hits (such as human albumin, bovine albumin, and murine alpha-fetoprotein).

LC-MS/MS showed that plasma albumin levels were 3.5 x 10^7^ ± 4.0 x 10^6^ (normalized precursor ion peak area referred to as label-free quantitation, LFQ) in Alb^-/-^ mice and 2.9 x 10^11^ ± 7.1 x 10^9^ (LFQ) in WT, with a fold difference of approximately 8,400 between these two genotypes (*P* < 0.0001, Fig 1). The extent of sequence coverage reflected the rigorous nature of this LC-MS/MS analysis of albumin (Fig 2). The analyzed peptides from the *in vitro* digestion of albumin, analyzed by this method, spanned 482 amino acids in WT mice, with 79% coverage of the protein’s amino acid sequence. Analysis of unique peptides further confirmed the robust depletion of albumin in Alb^-/-^ mice compared to WT. Fifty-eight unique peptides were identified in WT replicates whereas only 4 were identified in at least one of the Alb^-/-^ replicates. Specifically, over 30 unique peptides to murine albumin were identified in at least 50% of the WT replicates, with only one unique peptide identified in at least 50% of the Alb^-/-^ plasma samples. Additional analysis on this single unique peptide (LSQTFPNADFAEITK) that was identified in all mice in the study showed significantly lower levels in Alb^-/-^ than WT mice (Alb^-/-^, 1.7 x 10^7^ ± 4.9 x 10^5^; WT, 2.7 x 10^10^ ± 1.4 x 10^9^ LFQ, *P* < 0.001), further indicating a robust depletion of albumin in the Alb^-/-^ mice compared to WT (Fig 2A).

### Gel electrophoresis

**Fig 3:**
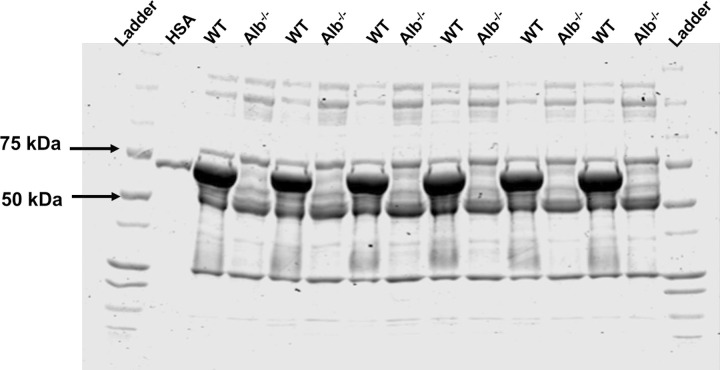
Coomassie staining of sodium dodecyl sulfate-polyacrylamide electrophoresis gel for plasma proteins in mice. Human serum albumin (HSA) and molecular weight markers were used as a reference. Alb^-/-^ mice showed a drastic reduction in protein abundance at the molecular weight of albumin (66.5 kDa), while WT mice presented a dark band at the same position.

SDS-PAGE with Coomassie staining demonstrated a drastic reduction of protein at the molecular weight of albumin (66.5 kDa) in Alb^-/-^ mice, while there was a comparatively darker band at albumin’s molecular weight in WT mice (Fig 3). A human albumin standard and molecular weight markers were used as the references for the result interpretation. The results from SDS-PAGE visually and qualitatively supported the findings from LC-MS/MS.

### BCG and BCP

The BCG assay moderately overestimated plasma albumin levels (Alb^-/-^: 1.2 ± 0.05 g/dL, WT: 3.7 ± 0.1 g/dL), as indicated by significant detection of apparent albumin levels in Alb^-/-^ plasma (Fig 1). The BCG kit was able to detect a statistically significant difference between WT and Alb^-/-^ mice (*P* < 0.0001). On the contrary, BCP greatly overestimated the plasma albumin concentration in both WT and Alb^-/-^, as demonstrated by the very high signal in Alb^-/-^ plasma (Fig 1). Furthermore, no significant difference was detected between these two genotypes when using BCP. To confirm the findings from the BCP assay, we also measured a subset of samples using a BCP kit from a second manufacturer. The results were similar (not shown). This excessive absorbance with BCP kits appeared to be the result of turbidity development, as discussed below. All samples were free from hemolysis or other potential artifacts that would affect the absorbance reading.

### ELISA

All plasma samples from Alb^-/-^ mice exhibited absorbances in the plate reader assay that were below that for the lowest albumin concentration in the calibration curve (i.e., albumin concentration below 0.0625 g/dL), and therefore albumin was considered ‘not detected’ in each of these samples. This observation supports the notion that albumin expression in this model is essentially negligible. ELISA estimated WT plasma albumin levels at 1.5 ± 0.1 g/dL (*P* < 0.0001). Unlike BCG and BCP assays, ELISA results agreed with LC-MS/MS and electrophoresis; samples known to have albumin expression very near zero indeed were characterized as analbuminemic by ELISA. All samples were free from hemolysis or other potential artifacts that would affect the absorbance reading.

## Discussion

Using the Alb^-/-^ mouse model as a negative biological control for plasma albumin testing, we showed that there was a moderate overestimation of albumin by BCG and a drastic overestimation by BCP. ELISA and LC-MS/MS appeared capable of properly characterizing analbuminemic plasma, and gel electrophoresis generated qualitative results in general agreement with ELISA and LC-MS/MS. From the present results, we infer that LC-MS/MS of trypsin-digests is a rigorous method against which other methods can be benchmarked. Overall, it was determined that ELISA, LC-MS/MS, and gel electrophoresis are capable of properly identifying analbuminemia. BCG and BCP should be used with caution as they could produce misleading results, particularly in cases with extreme albumin deficiency.

The overestimation of albumin concentration by the BCG assay has been well described in previous literature. One major reason for poor performance is non-specific binding between the BCG reagent and proteins other than albumin [22]. For example, globulins (e.g., α2-globulins) are among the plasma protein classes that can bind to BCG [3, 23]. Carbamylation of albumin, acute phase protein expression levels, and uremic toxins are also potential confounders of BCG dye performance that exhibit altered levels in various health conditions [12, 22, 24]. It is possible that this worsened performance of the assay also occurs in analbuminemic plasma as a moderately elevated α2-globulin expression is noted in CAA patients [25].

While BCP has been believed by some to exhibit specific binding to albumin, our current report indicates that such a claim must be reevaluated. We observed that the BCP assay very likely overestimated albumin concentration in WT mice and certainly overestimated albumin to a great extent in Alb^-/-^ plasma. Overestimation of albumin by the BCP assay has been reported previously as well, and it was suggested to be the result of the precipitation of fibrinogen, leading to turbidity which would interfere with light absorbance measurements [26]. In alignment with this report, we did consistently observe high turbidity in Alb^-/-^ samples after the addition of BCP reagents. It is possible that the performance of BCP is dependent on the characteristics of patients being tested (e.g., health conditions, genotypes or species). For example, BCP showed underestimated values in patients with renal insufficiency but was in good agreement with immunoassays when studying non-renal patients [27]. On the contrary, BCP has been purported to yield high accuracy in chronic kidney patients with all stages, including hemodialysis patients [6]. Nonetheless, it is a significant concern that unidentified substances accumulating in plasma in different patient populations could alter the behavior of BCP reagents [10, 11, 27]. The plasma proteome remodeling in both humans with CAA [25] and Alb^-/-^ mice [20, 21] is a concern in this regard. In both species, analbuminemia leads to higher expression of multiple proteins, including fibrinogen [20, 25], and this high fibrinogen expression may lead to turbidity and thus improper spectrophotometer performance. It should also be noted that in both BCG and BCP assays, bovine serum albumin was used as the standard for quantitation of samples. However, the assays have been tested for numerous species, according to the manufacturer. While it is possible that a mouse serum albumin standard would exhibit a modestly different response factor (i.e., a different standard curve slope), this is expected to be minimal because of the reasonably conserved nature of the albumin gene sequence in mammals. Furthermore, any differences in response factor between bovine and murine albumin should have a negligible impact on quantitation of albumin near values of zero (i.e., analysis of analbuminemic samples). That is to say, an analbuminemic sample, analyzed with a valid assay, should show an assay response value that is the same as the “blank” in the assay or the same as the y-intercept of the standard curve. This was not the case for dye-binding assays, indicating an error that is intrinsic to the behavior of the assay reagent. While the results reported here are expected to be related to the chemistry of BCG and BCP reagents, which would likely be similar between different vendors, it should be noted that in clinical settings the measurements of albumin are typically carried out using automated analyzers, and there might be some minor differences between instrument models and reagent manufacturers. Thus, future studies of testing Alb^-/-^ mouse samples in different clinical settings should be conducted.

The tendency for erroneous readings from dye-binding assays particularly impacts the diagnosis of CAA [28]. Not all the CAA reports specify their methods for estimating albumin levels, but in papers where methods were disclosed, we noted systematically higher albumin values from BCG and BCP assays in comparison to the other methods used in the same report [14–17, 28]. In case reports we have seen the serum albumin level in patients with CAA obtained by BCG and BCP assays typically range from 0.15–1.6 g/dL, and 0.8–1.6 g/dL, respectively [14, 16, 17, 29–32], while immunoassays yielded lower albumin values ranging from 0 to 0.2 g/dL [14, 16, 17, 33, 34]. However, CAA is a heterogeneous condition with different patients exhibiting different mutations; therefore, it cannot be stated that every CAA patient should exhibit exactly zero albumin expression. Thus, the comparison made above does not yet conclusively establish that immunoassay is superior to dye-binding assays for the detection of analbuminemia. Nonetheless, this observation can serve as a warning about the potential risk of using dye-binding assays when analbuminemia is a possibility. We used a more well-characterized biological model to represent analbuminemia, the Alb^-/-^ mouse, to take this hypothesis inferred from CAA patients and to rigorously test it. The LC-MS/MS analysis reported here verified the robust depletion of albumin in Alb^-/-^ mice through quantitation of this protein with high sequence coverage. Theoretically, one reason for disagreement between methods could be that truncated forms of albumin can be detected by only certain methods. Thus, it was critical to analyze the LC-MS/MS data in a way that could address this possible concern. The high sequence coverage revealed that the albumin measurement by LC-MS/MS was based upon the detection of the peptides collectively representing the majority of albumin’s amino acid sequence. Next, it was critical to identify if there was a specific fragment of albumin that might be circulating in Alb^-/-^ mice at high levels. Thus, we checked for unique peptides that were represented in both WT and Alb^-/-^ mice, and a single peptide (LSQTFPNADFAEITK) was identified. Even this sole peptide (generated from trypsin digestion of albumin *in vitro*) in Alb^-/-^ mouse plasma circulated at extremely low levels (over 1000-fold below levels in WT mice), further confirming that Alb^-/-^ mouse plasma can be considered as a reference material that is nearly albumin-free, and likely to be free of small fragments of the protein.

In this study, we employed untargeted LC-MS/MS, a method exhibiting high sensitivity and accurate detection of analbuminemia, compared to the other methods evaluated. To translate these results for clinical application and while further enhancing the precision, it could be beneficial in future studies to develop a targeted LC-MS/MS assay using Multiple Reaction Monitoring (MRM). An MRM assay may allow for even faster, more sensitive, and more accurate analysis compared to the untargeted approach. A targeted LC-MS/MS method in the future would involve selecting predetermined peptide(s) and optimizing LC-MS/MS conditions to monitor specific precursors and product ions associated with albumin. For patients in which very low levels of albumin are expected, LC-MS/MS could be considered in the future as a potentially useful assay in clinical pathology settings. Nonetheless, the use of LC-MS/MS in research here supports the validity of ELISA (and thus potentially other immunoassay approaches) which are less technically burdensome than mass spectrometry and could therefore be a very valuable tool for detecting analbuminemia.

In addition, it is noteworthy that the present findings have implications that may go beyond human medicine, with relevance to fields of veterinary pathology and comparative physiology. For example, while cartilaginous fish apparently express no albumin [35–37], widespread acceptance of this observation has been impeded by methodological issues. While it had been thought by some that cartilaginous fish actually do express albumin because of results from BCG assays, it was then shown that this albumin expression was not apparent when analyses were conducted by electrophoresis [36]. As indicated by electrophoresis results, it appears that cartilaginous fish simply evolved to express no albumin [36, 37]. Based upon our current results, it indeed appears that BCG assays are not reliable for determining if a species expresses albumin.

Evaluation of methods for the detection of analbuminemia is relevant to various fields of science, including comparative pathology, comparative physiology, evaluation of human rare diseases, as well as for proper characterization of laboratory mice for biomedical research. When characterization of very low or absent albumin expression is necessary, assay selection is critical. Overall, for identifying cases of analbuminemia, it is recommended here to use an immunoassay or LC-MS/MS, potentially in combination with electrophoresis. While analbuminemia is considered rare, one might wonder how many cases have been missed over the years because clinicians rely on ineffective dye-binding assays. BCG and BCP should be used with caution as they can produce misleading results. In veterinary patients from species for which immunoassay or LC-MS/MS will not be technically feasible (e.g., no available antibodies, no proteomics database for that species), it is suggested that an electrophoresis method will likely be more appropriate than a dye-binding assay.

## Conclusions

In conclusion, with the use of Alb^-/-^ mice as a negative biological control, we showed that BCG and BCP are inappropriate reagents for determining plasma albumin levels. LC-MS/MS and immunoassays like ELISA can accurately characterize albumin deficiency in analbuminemic organisms, with visual confirmation from electrophoresis. Specifically, LC-MS/MS was determined to be a rigorous and suitable method to test for extreme albumin deficiency. By using reliable assays, valid identification of analbuminemia in clinical practice can be achieved consistently in the future. Also, any missed diagnosis and false characterization of analbuminemia could be greatly reduced if the inaccurate albumin assay types are avoided in the future. Furthermore, it is also strongly recommended that authors in research studies and case reports disclose the type of methods used. This would help scientists interpret the implications of the findings and may assist with future standardization of the cutoff values for diagnosis and treatment of diseases.
